# Active Substances from *Callicarpa nudiflora* Exert Anti-Cervicitis Effects and Regulate NLRP3-Associated Inflammation

**DOI:** 10.3390/molecules26206217

**Published:** 2021-10-14

**Authors:** Tianchi Liu, Ruiqi Wang, Chenpeng Liu, Jiahong Lu, Yitao Wang, Lin Dong, Xiaopo Zhang

**Affiliations:** 1 State Key Laboratory of Quality Research in Chinese Medicine, Institute of Chinese Medical Sciences, University of Macau, Taipa, Macau 999078, China; YB77505@um.edu.mo (T.L.); Jiahonglu@um.edu.mo (J.L.); ytwang@um.edu.mo (Y.W.); 2 Key Laboratory of Tropical Translational Medicine of Ministry of Education, Hainan Provincial Key Laboratory for Research and Development of Tropical Herbs, School of Pharmacy, Hainan Medical University, Haikou 571199, China; hy0308018@hainmc.edu.cn (R.W.); hy0207042@hainmc.edu.cn (C.L.)

**Keywords:** *Callicarpa nudiflora*, active substances, anti-cervicitis, diterpenoids, NLRP3

## Abstract

Luohuazizhu suppository is a Traditional Chinese Medicine used in clinic to treat cervicitis, which is prepared from *Callicarpa nudiflora* Hook. et Arn (*C. nudiflora*), an herbal Chinese medicine named Luohuazizhu. This study aimed to figure out the active constituents of *C. nudiflora* and the potential mechanism for its anti-cervicitis effect. The ethanol extract in *C. nudiflora* (CNE) and the different fractions of CNE extracted by petroleum ether (CNE-p), dichloromethane (CNE-d), and n-butanol (CNE-b) were tested in vivo for their anti-cervicitis effects. Then the isolated compounds from the CNE-p were tested in vitro for their anti-inflammatory activities. The results displayed that CNE-p, CNE-d, and CNE-b exhibited adequate anti-cervicitis effects, with CNE-p showing the highest efficacy. Further experiment demonstrated that CNE-p could significantly inhibit the expression of NLRP3 in vitro. Six diterpenoids obtained from the CNE-p showed the ability to regulate inflammatory factor levels in vitro. Among these compounds, compounds **1** (callicarpic acid A) and **2** (syn-3,4-seco-12S-hydroxy-15,16-epoxy-4(18),8(17),3(16),14(15)-labdatetraen-3-oic acid) were the most effective agents, and they also inhibited the expression level of NLRP3 in vitro. The results confirmed that *C. nudiflora* has significant anti-cervicitis effects and the diterpenoids were most likely to be its active components. These data provide scientific support for the clinic usage of Luohuazizhu suppository and the development of new agents in treating cervicitis.

## 1. Introduction

Cervicitis is the inflammation of uterine cervix that mostly occurs in women of childbearing age. It is well-documented that cervicitis is usually caused by bacterial pathogens, including *staphylococci*, *streptococci*, and *anaerobes*, etc. [1]. Sometimes, cervicitis can also be caused by mechanical or chemical irritation, such as douching, etc. [2]. The symptoms of cervicitis are usually nonspecific but it is commonly accompanied with an increase in vaginal discharge and/or abdominal pain, intermenstrual bleeding [3]. For diagnosis, there are commercial systems based on molecular techniques that include almost all of the known pathogens associated with cervicitis, although cultures should not be abandoned due to the need to conduct studies of susceptibility to antibiotics. Levofloxacin, azithromycin, and other antibiotics are used in clinics to treat cervicitis [4]. Although progress has been made in the treatment of this disease, the effectiveness is still not satisfactory and drug resistance usually occurs [5]. Therefore, more effective and safe drugs in curing cervicitis are still needed in clinics.

Traditional Chinese Medicine (TCM) has potential effects in the treatment of cervicitis in clinic [6], for example, fukeqianjin formula [7], baofukang suppository [8], and XiaoMiShuan suppository (XMS) [9], etc. Luohuazizhu suppository is a Traditional Chinese Medicine used in clinic to treat cervicitis. This drug was prepared from *C. nudiflora*, an herbal Chinese medicine named Luohuazizhu [10]. *C. nudiflora* is a plant that can grow up to shrub or small tree and is mainly distributed in Hainan, Guangdong, and Guangxi Province, China [11]. As a medicinal plant, *C. nudiflora* has multiple pharmacological effects including anti-inflammatory, antibacterial, and hemostatic effects [12,13,14]. The extract of its aerial parts has been made into many clinical preparations such as Luohuazizhu tablet, Luohuazizhu capsule, Luohuazizhu suppository [15]. Animal experiments have shown that the extract of *C. nudiflora* is effective to ameliorate cervicitis by modulating inflammatory factors in rats [6]. The *C. nudiflora*-derived Luohuazizhu suppository is also effective in the clinic to treat cervicitis [16]. It helps to diminish cervical congestion and edema, alleviate mild cervical erosion, and improve vaginal discharge, characteristics and odor, congestion of the vaginal mucosa and other aspects [16,17,18]. Despite its significant clinical effect, the active substance and potential mechanism of *C. nudiflora* are still largely unknown. Identifying the active compounds of *C. nudiflora* and clarifying their potential mechanisms will be beneficial for its clinical use and development of new drugs for the treatment of cervicitis.

In this study, we established a phenol-induced cervicitis rats model and anRAW264.7 macrophage-based cell model to identify the active extracts and compounds of *C. nudiflora*. Interleukin-1*β* (IL-1*β*), interleukin-18 (IL-18), nuclear transcription factor-κB (NF-κB) and other inflammatory factors in serum, tissues, and cell culture supernatant were investigated. Diterpenoids from the active extract were tested for their anti-inflammatory activity. The regulatory effects of the active compounds on NLRP3 expression were also measured. 

## 2. Results

### 2.1. The Extracts Exhibited Low Cytotoxicity to RAW264.7 Cells

**Figure 1:**
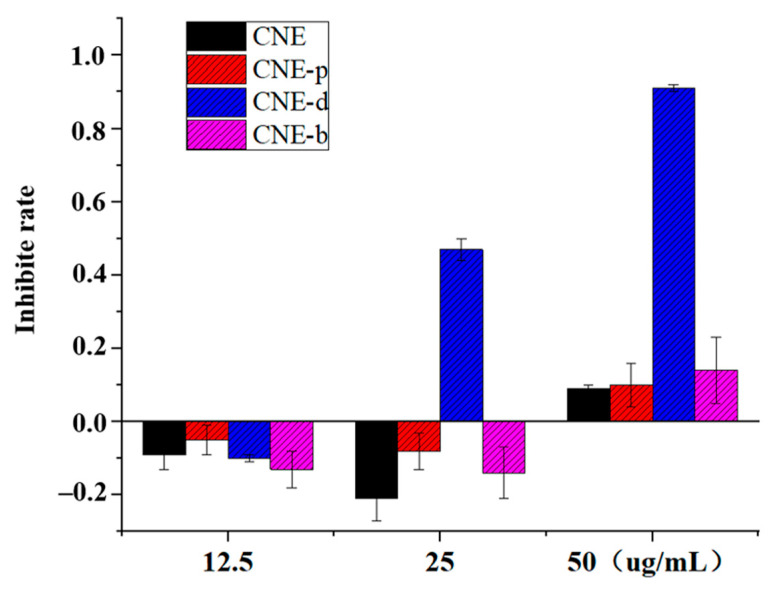
Cytotoxic effects of extracts obtained from *C. nudiflora* on RAW264.7 cells.

As shown in Figure 1, the cytotoxic effects of ethanol extract of *C. nudiflora* (CNE) and the extracts of petroleum ether extract (CNE-p), the dichloromethane extract (CNE-d), the n-butanol extract (CNE-b) obtained from CNE on RAW264.7 cells were not strong. Among these extracts, the rates of cell growth inhibition upon treatment with the CNE, CNE-p, and CNE-b components were approximately 10% at the dose of 50 µg/mL, which had little influence on the normal growth and reproduction of cells. The cytotoxicity of CNE-d was stronger, and the cell growth inhibition rate was less than 10% at 12.5 μg/mL. In summary, to avoid the influence of the cytotoxic effect of the sample itself on the assessments of inflammation and other related cytokines, the dosage of 50 μg/mL for CNE, CNE-p, and CNE-b was chosen for the following experiments. Meanwhile, the dosage of CNE-b was selected for 12.5 μg/mL. In addition, the cytotoxic effects of six diterpenoids were evaluated, and these compounds were found to exhibit no cytotoxicity.

### 2.2. Anti-Inflammatory Activity of the Extracts In Vitro

**Figure 2:**
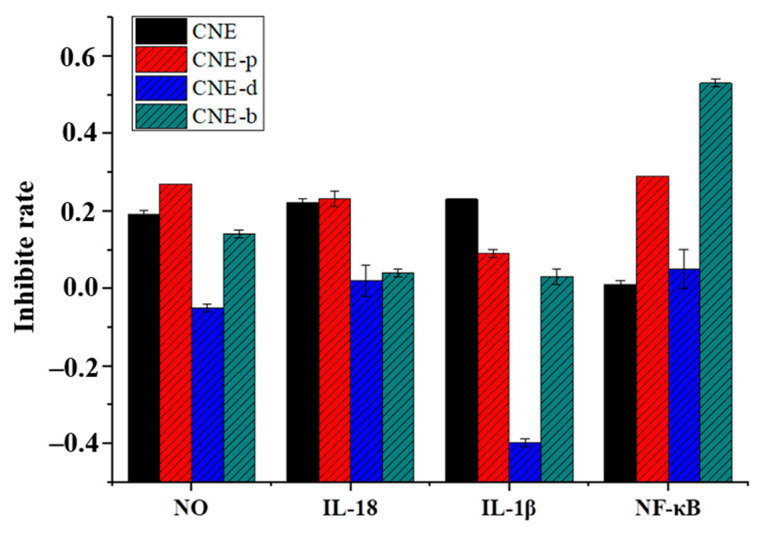
Influence of extracts obtained from *C. nudiflora* on changes in the inflammatory cytokines NO, IL-18, IL-1*β*, and NF-κB on RAW264.7 cells.

Combined with the research results, CNE-p showed a better effect on inhibiting nitric oxide (NO) production (Figure 2) with an inhibition rate of 27.27%. The LPS-induced RAW264.7 cell was used to evaluate the effects on three inflammatory factors of IL-1*β*, IL-18, and NF-κB. The results showed that CNE and CNE-p had more significant effects on IL-18, NF-κB. CNE-b inhibited NF-κB-dependent inflammatory cytokine release by up to 53.05%. These data suggested that CNE-p could inhibit all these inflammatory cytokines as shown in Figure 2. In summary, CNE, CNE-p showed relatively good anti-inflammatory activity. Furthermore, the regulation of NLRP3 by CNE-p was measured by ELISA in vitro. The results showed that CNE-p could inhibit the level of NLRP3 in LPS-injured cells and had a potential anti-inflammatory effect (Table 1).

### 2.3. The Extracts Affected the Body Weight Gain

**Figure 3:**
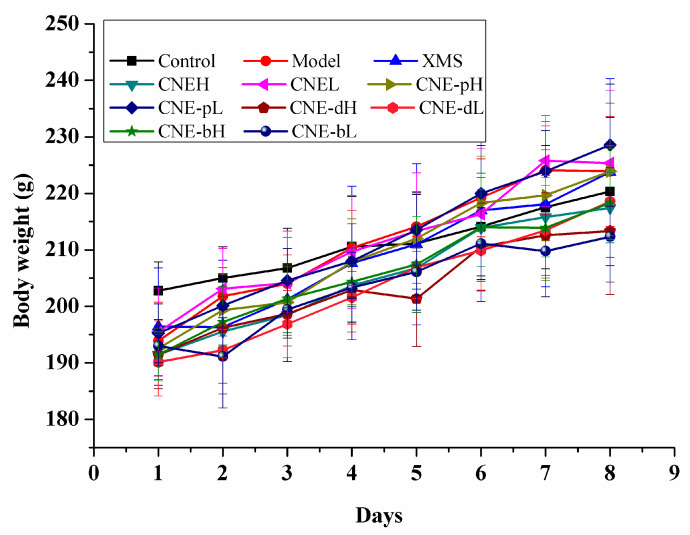
Body weight changes of SD rats treated with different extracts of *C. nudiflora*.

Compared with the control group, the rat’s body weights in the other groups all decreased, indicating that cervicitis caused damages to the normal health of rats as shown in Figure 3. After the treatment, the body weights of the rats in each group increased compared with the model group. The trends of body weight recovery in the CNEL (CNE low dosage), CNE-dL (CNE-d low dosage), and CNE-pH (CNE-p high dosage) groups during treatment were essentially the same as the positive group. These results showed that the above treatments displayed anti-cervicitis effects as shown in Figure 3. In addition, the body weight changes of the CNE-bH (CNE-b high dosage) and CNE-dH (CNE-d high dosage) groups were significantly decreased. However, the body weight was still lower than that of the model group after 8 days of treatment, indicating the potential toxic effects of the drugs in the above groups, as shown in Figure 3.

### 2.4. The Extracts Showed Significant Activity in Reducing Inflammation in Cervical Morphology

**Figure 4:**
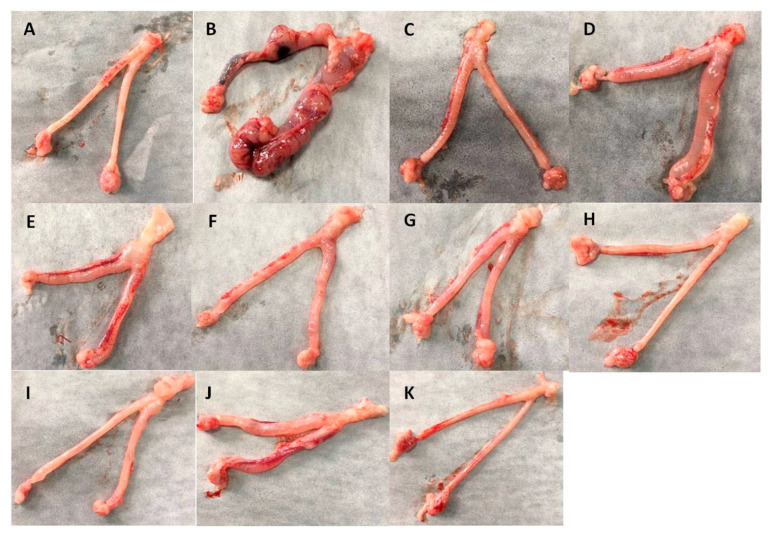
Morphological changes in the cervixes of SD rats treated with different extracts obtained from *C. nudiflora*. (**A**) Control group. (**B**) Model group. (**C**) XMS-treated group. (**D**) CNEH-treated group. (**E**) CNEL-treated group. (**F**) CNE-pH-treated group. (**G**) CNE-pL-treated group. (**H**) CNE-d H-treated group. (**I**) CNE-dL-treated group. (**J**) CNE-b H-treated group. (**K**) CNE-bL-treated group.

**Figure 5:**
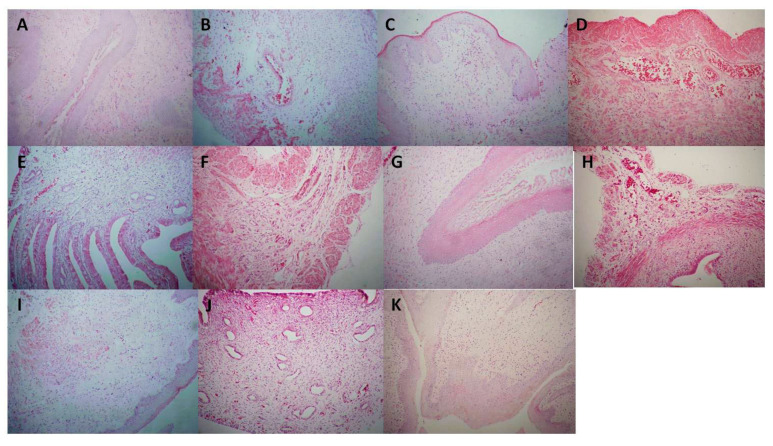
Histopathological changes in the cervixes of SD rats treated with different extracts obtained from *C. nudiflora*. (**A**) Control group. (**B**) Model group. (**C**) XMS-treated group. (**D**) CNEH-treated group. (**E**) CNEL-treated group. (**F**) CNE-p H-treated group. (**G**) CNE-pL -treated group. (**H**) CNE-dH-treated group. (**I**) CNE-dL-treated group. (**J**) CNE-bH-treated group. (**K**) CNE-bL-treated group.

Compared with the control group (Figure 4A), the model group had severe redness, swelling, obvious congestion, and erosion, but the related structural characteristics were not obvious (Figure 4B), indicating that the establishment of the cervicitis model was successful. In the XMS (XiaoMiShuan suppository) positive control group (Figure 4C), the redness and swelling of cervical tissues were significantly improved without hyperemia and erosion, indicating that the drug had a significant anti-cervicitis effect. Groups such as CNEH, CNEL (Figure 4D,E), CNE-pH and CNE-pL (Figure 4F,G), CNE-dH and CNE-dL (Figure 4H,I), and CNE-bH and CNE-bL (Figure 4J,K) were also evaluated. The results showed that CNE-p and CNE-d had significant therapeutic effects, with no significant hyperemia or erosion, as shown in Figure 4F–I. There was no significant difference in other indicators except for certain hepatomegaly, indicating that the extracts had no significant toxicity. Based on the above HE staining results, the cervical gland structures in the CNE-p, CNE-d, and CNE-b medium groups were intact and no significant hyperemia was found as shown in Figure 5. The treatment results were similar to those of the XMS group.

### 2.5. Changes in the Liver, Kidney, Spleen, Thymus and Other Organs Treatment with Different Extracts

**Figure 6:**
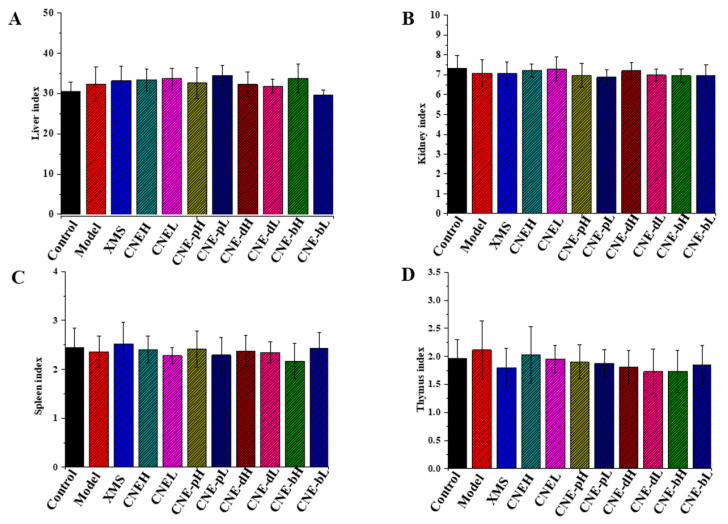
Organ index changes of SD rats treated with different extracts of *C. nudiflora*. (**A**) Liver. (**B**) Kidney. (**C**) Spleen. (**D**) Thymus.

The organ index refers to the ratio of the organ weight to body weight. The results indicated that the extracts have little influence on the organ indexes of the liver, kidney, spleen, thymus and other organs in rats as shown in Figure 6. These extracts are safe for the organs of rats.

### 2.6. Inflammatory Cytokine Changes in Cervical Tissues and Serum Treated with Different Extracts

**Figure 7:**
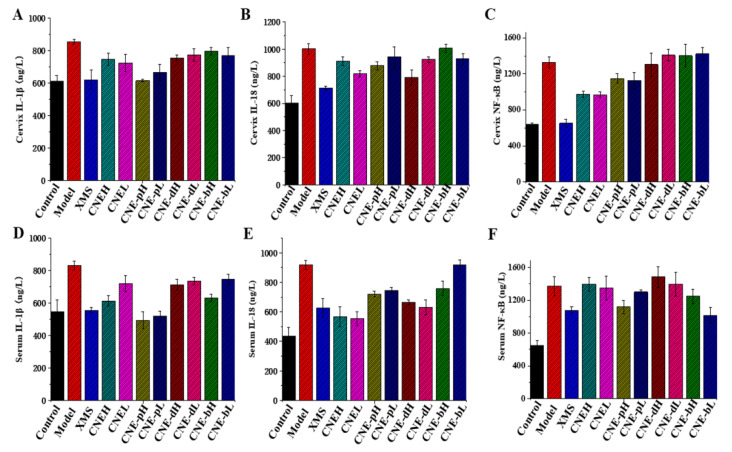
Changes of inflammatory factors in SD rats treated with different extracts obtained from *C. nudiflora* (CNE, CNE-p, CNE-d, CNE-b). The levels of inflammatory factors in the cervix and serum were detected by ELISA. (**A**) Changes in cervical IL-1*β* levels after treatments with different extracts. (**B**) Changes in cervical IL-18 levels after treatments with different extracts. (**C**) Changes in cervical NF-κB expression after treatments with different extracts. (**D**) Changes in serum IL-1*β* levels after treatments with different extracts. (**E**) Changes in serum IL-18 levels after treating with different extracts. (**F**) Changes in serum NF-κB levels after treating with different extracts.

In combination with the ELISA results, the levels of IL-1*β* in serum and cervical tissues were significantly inhibited in the CNE-p group, and the effect was similar to the XMS group. The levels of IL-18 in cervical tissues were improved in the CNEL and CNE-pH groups, and the serum IL-18 levels were significantly reduced in the CNE groups. The CNE treatment also reduced the expression level of NF-κB in cervical tissue, and the CNE-pH and CNE-b treatments alleviated the elevated levels of serum NF-κB. In conclusion, the CNE-p showed inhibitory effects on the levels of IL-1*β*, IL-18, NF-κB and other inflammatory factors. In the CNE group, inflammatory factors such as IL-18 and NF-κB were significantly alleviated, as shown in Figure 7. These results showed that CNE and CNE-p showed significant therapeutic effects on cervicitis, and both of them showed activities in inhibiting the levels of cytokines. Therefore, this study firstly demonstrated that CNE had anti-inflammatory effects both in vivo and in vitro. CNE-p is the active fraction obtained from CNE.

### 2.7. Anti-Inflammatory Activity of Diterpenoids from CNE-p In Vitro

**Figure 8:**
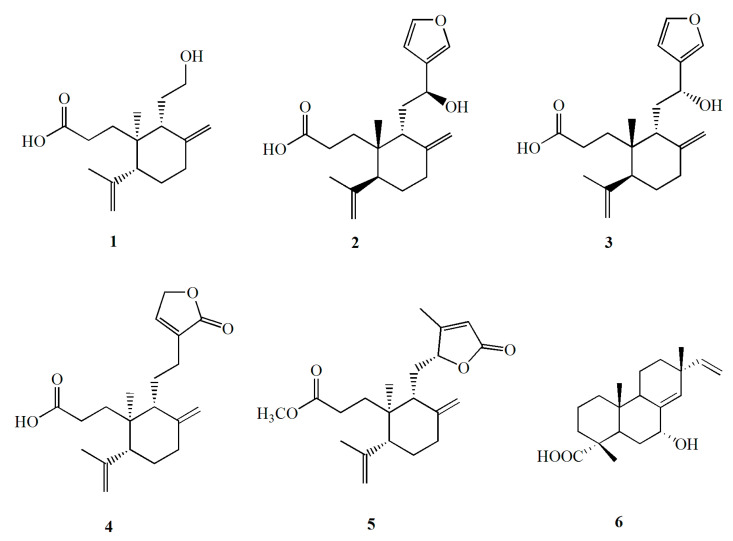
Chemical structures of six diterpenoids obtained from CNE-p.

**Figure 9:**
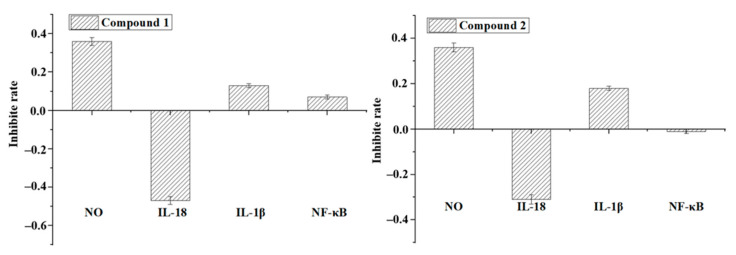
Anti-inflammatory activities of compounds **1** and **2** obtained from CNE-p.

By chemical investigations, six diterpenoids were obtained and structurally identified (Figure 8). To confirm whether these compounds possess anti-inflammatory activity, all of them were tested in vitro. The results revealed that the compounds also showed anti-inflammatory activity. Both compounds **1** (callicarpic acid A) and **2** (syn-3,4-seco-12S-hydroxy-15,16-epoxy-4(18),8(17),3(16),14(15)-labdatetraen-3-oic acid) effectively decreased the secretin of NO and IL-1β but stimulated IL-18 (Figure 9). For the key pro-inflammatory factor of NF-κB, compound **1** markedly inhibited its production but compound **2** showed little effect (Figure 9). 

### 2.8. The Binding Energy and the Amino Acid Residue between Compounds and NLRP3

**Figure 10:**
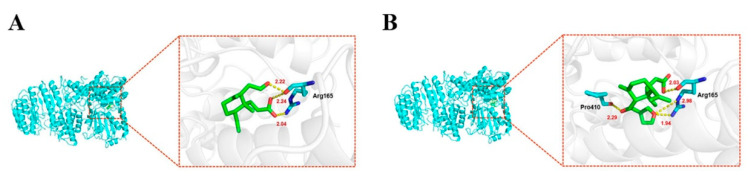
Docking and binding pattern of compounds **1** (**A**) and **2** (**B**) with into NLRP3 active site.

In the molecular docking study, the docked structure of compound **1** with NLRP3 exhibited a binding energy of −1.86 kcal/mol. Docking results showed that compound **1** could bind with amino acid residue Arg165. The hydrogen atom of the carboxylic acid group and hydrogen atom of the hydroxyl group in compound **1** formed two hydrogen binds each with amino acid residues Arg165. The oxygen on the carbonyl group of the carboxylic acid group formed the hydrogen bond with amino acid residues ARG165. While compound **2** exhibited a binding energy of −2.03 kcal/mol with NLRP3, docking study has shown that compound **2** could bind with two amino acid residue, such as Arg165 and PRO410. The hydrogen atom of the carboxylic acid group of compound **2** formed hydrogen bond with amino acid residues Arg165. The oxygen atoms in the furan ring formed two hydrogen bond with amino acid residues ARG165.The hydrogen atom of the hydroxyl group formed the hydrogen bond with amino acid residues PRO410 (Figure 10).

### 2.9. Regulating Effects of Diterpenoids on NLRP3 In Vitro

After LPS injury, the expression of NLRP3 in cells increased significantly compared with the control. Compounds **1** and **2** inhibited the level of NLRP3 in LPS-damaged cells in a dose-dependent manner. Notably, the level of NLRP3 in the compound **2** was lower than that in the normal group, indicating that the compounds had a good anti-inflammatory effect. The above results indicate that compounds **1** and **2** could regulate the level of NLRP3 in vitro (Table 1). By these investigations, the diterpenoids in CNE-p, especially compound **2**, displayed anti-inflammatory activity.

### 2.10. Protein Expressions Regulating Effects of Compounds **1** and **2** on NLRP3

**Figure 11:**
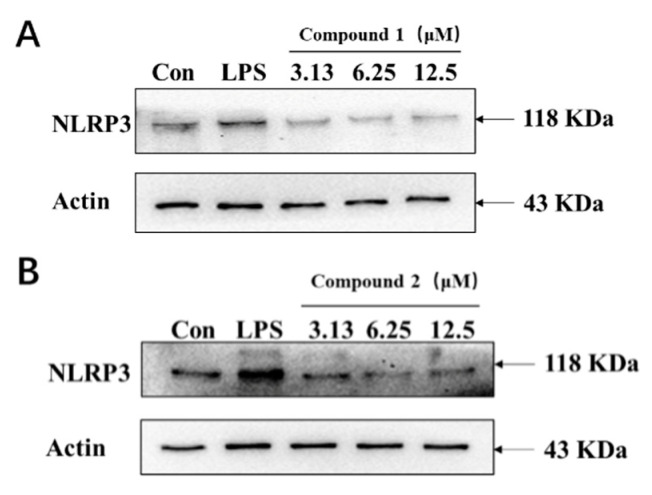
Inhibitory effects of compounds **1** and **2** on the NLRP3 expression in vitro. (**A**) The expression of NLRP3 after treatment with three doses of compound **1**. (**B**) The expression of NLRP3 after treatment with three doses of compound **2**.

Western blots were used to analyze the levels of NALP3 in LPS-induced inflammatory cell models, and the results showed that both compounds **1** and **2** could dose-dependent reverse the LPS-induced high expression of NLRP3 by Western blot. Therefore, both compounds have potential anti-inflammatory effects, but the specific mechanism needs to be further investigated (Figure 11).

## 3. Discussion

Cervicitis is a common gynecological disease that threatens women’s health and seriously worsens the quality of their life [19,20]. Clinical reports have pointed out that, in women with chronic cervicitis and chronic pelvic inflammatory disease, ovarian and fallopian tube dysfunction can lead to serious adverse pregnancy outcomes and infertility. Moreover, chronic cervicitis also increases the incidence of cervical cancer [21,22]. Therefore, effective treatment for cervicitis is of great significance for improving patients’ life quality, gestating new life, and preventing cervical cancer. Although current therapy with antibiotics can achieve certain clinical benefits, the recurrence rate remains high, and the long-term effect is not satisfied. Traditional Chinese medicine (TCM) is a good alternative to antibiotics for the treatment of cervicitis, which has been used for thousands of years. Compared with antibiotics, TCMs turn on or off gene expression in different tissues to restore the morphology and physiological function. TCM treatment does not affect human cell components and shows less toxic side effects, which is especially important for patients for whom fertility is a priority [16].

Luohuazizhu suppository is made from *C. nudiflora*, an herbal Chinese medicine named Luohuazizhu [10]. Previous studies have shown that *C. nudiflora* has a strong inhibitory effect on bacterial and fungal diseases in clinical [12]. It is effective to improve microcirculation, increase local blood flow, promote inflammation rehabilitation, promote epithelial regeneration and repair diseased tissue [12,13,14], displaying adequate anti-cervicitis effects. However, the active substances of *C. nudiflora* have not been clarified up till now. In this study, we first identified the active fraction of *C. nudiflora* in phenol-induced cervicitis rats. Body weight gain, histology of cervical tissue, and inflammatory cytokines were main indicators in this work to evaluate the pharmacological effectiveness of different extracts of *C. nudiflora*. In general, low-polarity fractions of *C. nudiflora* showed higher efficiency than that with high-polarity in maintaining body weight, restoring morphology of cervical tissue, and decreasing inflammatory state. The petroleum ether fraction of the ethanol extract of *C. nudiflora* (CNE-p) exhibited the best therapeutic effect. Through these investigations, we demonstrated that the extract of *C. nudiflora* had significant anti-cervicitis effects for the first time. By a further step, the most active extract was firstly determined to be CNE-p which was the lipid soluble extract of *C. nudiflora*. Therefore, CNE-p is the active fraction obtained from *C. nudiflora*, and the active compounds should be with low-polarity.

We further isolated the compounds from CNE-p by column chromatography including silica gel, HPLC, etc., and six diterpenoids were obtained and structurally characterized. All the compounds were tested for their anti-inflammatory activities in vitro to identify the active components of *C. nudiflora*. The results showed that compounds **1** and **2** exhibited the best anti-inflammatory activities. Recently, Wang et al. [14] also isolated several diterpenoids from the leaves of *C. nudiflora* which displayed potent anti-inflammatory effects. These results indicated that diterpenoids might be the active substances of *C. nudiflora.*

The NLRP3 inflammasome is a multimeric protein complex that initiates an inflammatory form of cell death and triggers the release of proinflammatory cytokines IL-1*β* and IL-18. The NLRP3 inflammasome is involved in inflammation and the immune response in the body [23,24]. Certain agents can inhibit NLRP3, which has important effects on various inflammatory factors [25,26]. Many natural products had the ability in inhibiting NLRP3 [27]. By this study, the result showed that petroleum ether extract significantly regulated the level of NLRP3, especially compounds **1** (callicarpic acid A) and **2** (syn-3,4-seco-12S-hydroxy-15,16-epoxy-4(18),8(17),3(16),14(15)-labdatetraen-3-oic acid), could dose-dependently inhibit the level of NLRP3 in LPS-damaged cells. More significantly, the levels of NLRP3 in the two treated groups were lower than those in the normal group, indicating that they had good anti-inflammatory effects. These results showed that inhibiting the NLRP3 might be the potential mechanism for these compounds.

Previous studies regarding Luohuazizhu suppository mostly focus on its clinical anti-cervicitis effects [17,18]. Although, the effects have been confirmed in vitro [6]. The active extracts or compounds and the potential mechanisms are still unknown. By our investigations, we firstly clarified the anti-cervicitis effects of *C. nudiflora* and found the potential active diterpenoids. Diterpenoids are the characteristic compounds of *C. nudiflora*, and many of them showed anti-inflammatory activities by inhibiting NO production [10]. Apart from NO, we demonstrated that these compounds could inhibit other inflammatory factors and NLRP3 for the first time. Meanwhile, the potential mechanism of the active was also investigated. These results provide scientific supports for the clinic usage and drug development of Luohuazizhu suppository. In the future, obtaining more diterpenoids, structure-activity relationship studies, detailed mechanistic studies, and total synthesis studies, should be carried out to find more effective and safe agents from *C. nudiflora* for treating cervicitis.

## 4. Experimental

### 4.1. Plant Materials

The aerial parts of *C. nudiflora* were collected in Hainan Province of China (October 2018). The plant was identified by Prof. Niankai Zeng. A voucher specimen (No. CN201810) has been deposited in the School of Pharmacy, Hainan Medical University.

### 4.2. Extraction and Isolation

The dried aerial parts (5.0 kg) were powdered and extracted by ethanol under reflux for three hours. The extract was concentrated under reduced pressure to yield the ethanol extract (350.0 g) and was named CNE. The CNE (175.0 g) was dissolved in water (2.0 L) and was partitioned by petroleum ether (1.0 L), dichloromethane (1.0 L), and n-butanol (1.0 L) successively to yield an petroleum ether extract (35.0 g, CNE-p), a dichloromethane extract (23.0 g, CNE-d), and an n-butanol extract (66.0 g, CNE-b). CNE-p was isolated by silica gel column chromatography using a gradient ratio of petroleum ether-acetone as the eluent to yield some fractions. The fractions were further purified by applying column chromatography of ODS followed by semi-preparative HPLC to yield callicarpic acid A (**1**, 23 mg), syn-3,4-seco-12S-hydroxy-15,16-epoxy-4(18),8(17),3(16),14(15)-labdatetraen-3-oic acid (**2**, 15 mg), syn-3,4-seco-12R-hydroxy-15,16-epoxy-4(18),8(17),13(16),14(15)-labdatetraen-3-oic acid (**3**, 10 mg), ent-3,4-seco-14-carbonyl-15,16-epoxy-4(18),8(17),13(14)-labdatrien-3-oic acid (**4**, 7 mg), ent-3,4-seco-12R,15-epoxy-4(18),8(17),13(14)-labdatrihydroxy-3-oic acid (**5**, 8 mg), and 7α-hydroxy sandaracopimaric acid (**6**, 10 mg). All of the compounds were identified by comparing their NMR spectroscopic data [28,29,30].

### 4.3. MTT Assay

RAW264.7 cells were resuspended, passaged in RPMI 1640 culture medium containing 10% fetal bovine serum, seeded in a 50-mL culture flask, and cultured at 37 °C in a carbon dioxide incubator with 5% CO_2_. Cells in the logarithmic growth phase were diluted to 1 × 10^5^/mL and seeded into 96-well plates. Corresponding doses of extracts and compounds were given at the same time. After the treatments, the media were removed, and the cells were incubated with 50 μL of MTT solution for 4 h. The level of MTT formazan was determined by measuring its absorbance at 490 nm with a microplate reader.

### 4.4. Anti-Inflammatory Activity Test

The RAW264.7 cells were resuspended, passaged in RPMI 1640 culture medium containing 10% fetal bovine serum, and seeded in a 50 mL culture flask. Then, cells in the logarithmic growth phase were then diluted to 1 × 10^5^/mL and inoculated into 96-well plates. After 24 h culture, the cells were treated with 1 mg/mL LPS for 4 h, and then, the extracts and compounds were added to the treatment for 2 h, after which the superfluid from the medium was taken and tested according to the requirements of relevant kits.

### 4.5. Molecular Docking Studies

The X-ray crystal structure of NLRP3 (PDB ID code 6NPY) [31] was obtained from RCSB Protein Data Bank (www.rcsb.org/pdb) in 3 February 2021, water molecular was removed, and hydrogen atoms were added with the Autodock tools. The docking was performed by Autodock 4.2 with Lamarckian genetic algorithm to sift the best ligand-receptor interaction [32,33]. Finally, graphical representations were completed by PyMOL molecular graphics system (version 2.5 Schrödinger, LLC.) [34].

### 4.6. Western Blot Assay

After treatment, total proteins were extracted from cells, quantified, and subjected to 10% sodium dodecyl sulfate-polyacrylamide gel electrophoresis (SDS-PAGE); the bands were electrically transferred to PVDF membranes. After blocking, the membranes were probed with specific monoclonal antibodies against the target proteins. After washing the membrane and incubation with secondary antibodies, the signals were developed using an ECL kit. The NLRP3 and β-actin levels were detected by scanning and quantifying the blots. The expression levels of target proteins are presented as the fold change relative to the control treatment cells.

### 4.7. Animals and Experimental Design

All the animal experiments were performed in accordance with the National Institutes of Health regulations for the care and use of animals in research. The experimental protocols were approved by the Medical Ethics Committee of Hainan Medical University (No. SYXK-2017-0013). To evaluate the anti-cervicitis activity of different extracts of *C. nudiflora*, 110 female SD rats were purchased from Chengdu Dashuo Experimental Animal Co. Ltd. GemPharmatech Co., Ltd. (Chengdu, China). The animals were housed in a humidity-controlled room with a 12 h light-dark cycle and allowed free access to common food and water a week for acclimatization. An amount of 25% Phenol mucilage was prepared by mixing 1 g Arabic gum, 5 mL phenol, 4 mL glycerin, and 20 mL distilled water together. The control group was received the same dosage of phenol-free matrix, while the other rats were injected with 25% phenol mucilage at a dose of 0.1 mL·100 g^−1^ in the vagina by syringe seven times once every days to induce cervicitis. When the SD rats exhibited white secretions, red and swollen vaginas and vaginal hyperemia, the model was considered to be successfully established. Then, the model rats were randomly divided into ten groups: a model group, a positive control group, CNEH: a CNE high-dose group; CNEL: a CNE low-dose group; CNE-pH: a CNE-p high-dose group; CNE-pL: a CNE-p low-dose group; CNE-dH: a CNE-d high-dose group; CNE-dL: a CNE-d low-dose group; CNE-bH: a CNE-b high-dose group; and CNE-bL: a CNE-b low-dose group. Extracts were administered for 8 consecutive days. On the 8th night, the rats were fasted and could not stop drinking water. On the 9th day, tissues were harvested in the morning, and serum was prepared. During the experiment, the cervical lesions of the rats were observed and recorded every day, and weight changes were also detected. The relevant ELISA kits were carried out in accordance with the instructions.

The dose and drug administration method were designed as follows: high dose, 850 mg/Kg (suppository 600 mg/Kg); low dose raw material, 425 mg/Kg (suppository 300 mg/Kg); positive drug, 300 mg/Kg; the final dose was 100 μL/200 g; Control: blank Control group; Model: Model group; XMS: XiaoMiShuan suppository positive Control group; CNEH: CNE high-dose group; CNEL: CNE low-dose group; CNE-pH: CNE-p high-dose group; CNE-pL: CNE-p low-dose group; CNE-dH: CNE-d high-dose group; CNE-dL: CNE-d low-dose group; CNE-dH: CNE-b high-dose group; and CNE-bL: CNE-b low-dose group. The dose regimens were as follows: Blank group: Blank matrix; Model group: modeling plus blank matrix; Positive drug: modeling plus XiaoMiShuan suppository; and Sample set: modeling plus sample.

### 4.8. Statistical Analysis

For the in vitro results, the values represent the mean ± SEM of at least three separate experiments. For the animal experiments, the values represent the mean ± SEM of 8 mice in each group. GraphPad Prism 6 software was used for statistical analysis. After validation of the test for homogeneity of variance, differences among the studied groups were examined by one-way ANOVA followed by multiple comparisons.

## 5. Conclusions

In conclusion, based on the significant clinic effects of Luohuazizhu suppository in treating cervicitis, we aimed to find out its active compounds and potential mechanism. Then we designed a series of studies for the cytotoxic, anti-cervicitis, anti-inflammatory, and changes of inflammatory factors as well as NLRP3. By these studies, for the first time, we confirmed the significant anti-cervicitis effect of the ethanol extract of *C. nudiflora* at the animal level and found that the most significant effect was achieved by the petroleum ether extract (CNE-p). The diterpenoids contained in the extract were most likely to be its effective components. This class of ingredients may mediate their function by regulating the expression of inflammatory factors, especially NLRP3. The significant anti-cervicitis effects of CNE, CNE-p from *C. nudiflora* provided important pharmacology data for supporting the usage of Luohuazizhu suppository in clinic. Moreover, the active diterpenoids and the regulation of NLRP3 are important for finding new agents in treating cervicitis.

## Figures and Tables

**Table 1:** Influence of CNE-p, compounds **1** and **2** on changes of NLRP3 in LPS-injured RAW264.7 cells (*n* = 4).

Group	Concentration	NLRP3 (pg/mL)	Group	Concentration	NLRP3 (pg/mL)
Control	——	344.58	Compound **1**	3.125 μM	297.71 **##
LPS (1 mg/L)	——	517.50 **		6.25 μM	231.56 **##
				12.5 μM	202.40 **##
CNE-p	12.5 μg/mL	387.81 **##	Compound **2**	6.25 μM	432.08 **##
	25 μg/mL	363.85 ##		12.5 μM	416.46 **##
	50 μg/mL	357.60 ##		25 μM	383.13 **##

Compared with the control group, ** *p* < 0.01; compared with the LPS group, ## *p* < 0.01.

## Data Availability

The datasets used during the current study are available from the corresponding author upon reasonable request.

## Sample Availability

Samples of the extracts and compounds are available from the authors.
